# Evolution of internal variables in an expanding hollow cylinder at large plastic strains

**DOI:** 10.1186/s40064-016-2027-6

**Published:** 2016-03-29

**Authors:** Sergei Alexandrov, Nguyen Dinh Kien, Yaroslav Erisov, Fedor Grechnikov

**Affiliations:** Institute for Problems in Mechanics, Russian Academy of Sciences, 101-1 Prospect Vernadskogo, Moscow, Russia 119526; Institute of Mechanics, VAST, 18 Hoang Quoc Viet, Hanoi, Vietnam; Samara State Aerospace University, 34 Moskovskoe shosse, Samara, Russia 443086

**Keywords:** Internal variables, Hollow cylinder, Expansion, Large strains, Lagrangian coordinates, Rigid plastic material, Finite difference method

## Abstract

An efficient method for calculating the evolution of internal variables in an expanding hollow cylinder of rigid/plastic material is proposed. The conventional constitutive equations for rigid plastic, hardening material are supplemented with quite an arbitrary set of evolution laws for internal variables assuming that the material is incompressible. No restriction is imposed on the hardening law. The problem is solved in Lagrangian coordinates. This significantly facilitates a numerical treatment of the problem. In particular, the initial/boundary value problem is reduced to a system of equations in characteristic coordinates. A finite difference scheme is used for solving these equations. An illustrative example is presented assuming that the internal variables are the equivalent plastic strain and a damage parameter.

## Background

Expansion of a hollow cylinder is one of the classical problems of elasticity and plasticity. The first strict elastic/plastic solution for the expansion of a hollow cylinder of elastic/plastic material at large strains has been provided by Hill et al. (1947). Subsequent limit analysis has been used by Leu (2007, 2009), Leu and Li (2012) to find solutions for several rigid plastic models. A constitutive equation for nonlinear viscoelasticity has been adopted by Wineman and Min (1996). The location of yield in rotating thick-walled cylindrical shells made of functionally graded materials has been determined by Fatehi and Nejad (2014). A great number of solutions at small elastic/plastic strains have been proposed for various material models (see, for example, Bland 1956; Chen 1986; Megahed and Abbas 1991; Rees 1987, 1990; Stacey and Webster 1988; Lazzarin and Livieri 1997; Livieri and Lazzarin 2002; Loghman and Wahab 1994; Farrahi et al. 2013). However, in many cases elasticity is not so important at large strains, i.e. the elastic portion of the strain rate tensor may be neglected. In particular, rigid plastic models are used even in conjunction with finite element methods (see, for example, Guo and Kamitani 2010; Cheong et al. 2014; Eom et al. 2014). On the other hand, it is important to predict the evolution of internal variables since these variables control material properties. A typical model capable of such predictions is based on the conventional system of equations of plasticity theory (i.e. a yield criterion and its associated flow rule) and evolution equations for internal variables. These equations should be solved simultaneously. Commonly used internal variables are the equivalent strain, various damage parameters (Lemaitre 1985; Chandrakanth and Pandey 1993; Besson 2010 among many others), dislocation density (Ganapathysubramanian and Zabaras 2004; Lin and Dean 2005; He et al. 2012; Hore et al. 2015) and many other parameters that characterize the microstructure of material.

It is shown in the present paper that for a generic set of evolution equations for internal variables the initial/boundary value problem for an expanding hollow cylinder is reduced to a hyperbolic system of equations. The characteristic curves of this system are coordinate lines of a Lagrangian coordinate system. Therefore, a very high accuracy of numerical solutions can be easily achieved using a finite difference method and these solutions can serve as simple benchmark tests for numerical packages that deal with the prediction of the evolution of internal variables in metal forming processes. A necessity of such tests has been pointed out by Roberts et al. (1992). Note that even in the case of linear elasticity numerical results often depend on a particular method adopted to solve the problem (Helsing and Jonsson 2002). Therefore, reliable benchmark tests are important for large strain plasticity models that include internal variables.

The method proposed is an extension of the method developed in Alexandrov and Jeng (2011). In this paper, the evolution of damage in an expanding/contracting hollow sphere has been investigated.

## Statement of the problem

Consider a long thick-walled tube of initial outer radius $b_{0}$ and initial inner radius $a_{0}$. The tube is subject of uniform pressure $p_{0}$ applied over its inner radius. It is assumed that the magnitude of $p_{0}$ is high enough to initiate plastic yielding everywhere in the tube. Since the model adopted is rigid plastic, the magnitude of $p_{0}$ is determined from the solution. The current inner and outer radii of the tube will be denoted by $a_{c}$ and $b_{c}$, respectively. It is convenient to introduce a cylindrical coordinate system $\left( {r,\theta ,z} \right)$ with its z-axis coinciding with the axis of symmetry of the tube. The state of strain is two-dimensional in this coordinate system $(\varepsilon_{z} = 0)$. Here $\varepsilon_{z}$ is the axial strain. The initial/boundary value problem is axisymmetric and its solution is independent of $\theta$. The circumferential velocity vanishes everywhere. The normal stresses in the cylindrical coordinate system are the principal stresses. The stress boundary condition is

1 $$\sigma_{r} = 0$$

for $r = b_{c}$. Here $\sigma_{r}$ is the radial stress ($\sigma_{\theta }$ will stand for the circumferential stress). Models of rate-independent plasticity will be considered in the present paper. Therefore, it is possible, with no loss of generality, to arbitrarily prescribe the radial velocity at $r = b_{c}$. It is convenient to put

2 $$u = u_{0}$$

for $r = b_{c}$. Here *u* is the radial velocity and $u_{0}$ is constant.

According to a widely used model of hardening plasticity the yield stress depends on the equivalent strain, $\varepsilon_{eq}$, and the latter is defined by the following equation

3 $$\frac{{d\varepsilon_{eq} }}{dt} = \xi_{eq} .$$

Here *t* is the time, ${d \mathord{\left/ {\vphantom {d {dt}}} \right. \kern-0pt} {dt}}$ denotes the convected derivative and $\xi_{eq}$ is the equivalent strain rate. In the case under consideration, the latter is expressed in terms of the physical components of the strain rate tensor in the cylindrical coordinate system as

4 $$\xi_{eq} = \sqrt {\frac{2}{3}} \sqrt {\xi_{r}^{2} + \xi_{\theta }^{2} } ,$$

where $\xi_{r}$ and $\xi_{\theta }$ are the radial and circumferential strain rates, respectively. It has been taken into account here that the axial strain rate vanishes everywhere since $\varepsilon_{z} = 0$. The constitutive equations include the yield criterion, its associated flow rule and internal variable evolution equations. The von Mises yield condition modified to account for the effect of internal variables on the yield stress is written as

5 $$\sigma_{eq} = \sigma_{0} {\Phi} \left( {\varepsilon_{eq} } \right)\Lambda \left( {\alpha_{1} , \ldots ,\alpha_{k} } \right),$$

where $$\sigma_{eq}$$ is the equivalent stress, $$\sigma_{0}$$ is a reference stress, $$\alpha_{p}$$ are internal variables other than $$\varepsilon_{eq}$$$$(1 \le p \le k),$$$${\Phi} \left( {\varepsilon_{eq} } \right)$$ is an arbitrary function of its argument satisfying the conditions $${\Phi} \left( 0 \right) = 1$$ and $${{d{\Phi} } \mathord{\left/ {\vphantom {{d{\Phi} } {d\varepsilon_{eq} }}} \right. \kern-0pt} {d\varepsilon_{eq} }} \ge 0$$ for all $$\varepsilon_{eq}$$. In the case under consideration, the equivalent stress is defined by

6 $$\sigma_{eq} = \frac{\sqrt 3 }{2}\left( {\sigma_{\theta } - \sigma_{r} } \right).$$

It has been taken into account here that $\sigma_{\theta } > \sigma_{r}$ in the case of tube expansion. The associated flow rule reduces to

7 $$\xi_{\theta } = \lambda ,\quad \xi_{r} = - \lambda .$$

Here $\lambda$ is a non-negative multiplier. The magnitude of $\lambda$ is not important for applications. It is evident that $\xi_{\theta } > 0$ in the boundary value problem under consideration. Therefore, the inequality $\lambda > 0$ is satisfied and Eq. (7) reduces to the equation of incompressibility

8 $$\xi_{r} + \xi_{\theta } = 0.$$

Using this equation, it is possible to transform Eq. (4) to

9 $$\xi_{eq} = \frac{2}{\sqrt 3 }\xi_{\theta } .$$

It has been taken into account here that $\xi_{\theta } > 0.$ A typical evolution equation for internal variables can be written in the form (Lemaitre 1985; Chandrakanth and Pandey 1993; Ganapathysubramanian and Zabaras 2004; Lin and Dean 2005; Besson 2010; He et al. 2012; Hore et al. 2015)

10 $$\frac{{d\alpha_{p} }}{dt} = F\left( {\frac{\sigma }{{\sigma_{eq} }},\;\varepsilon_{eq} ,\;\alpha_{1} , \ldots ,\alpha_{k} } \right)\xi_{eq} ,\quad 1 \le p \le k,$$

where $\sigma$ is the hydrostatic stress and *F* is quite an arbitrary function of its arguments.

In the case under consideration the hydrostatic stress is defined as

11 $$\sigma = \frac{{\sigma_{r} + \sigma_{\theta } }}{2}.$$

The initial conditions to Eqs. (3) and (10) are

12 $$\varepsilon_{eq} = 0$$

and

13 $$\alpha_{p} = \alpha_{p0} ,\quad 1 \le p \le k$$

for $t = 0$ or $b_{c} = b_{0}$. The constitutive equations are supplemented by the only non-trivial equilibrium equation of the form

14 $$\frac{{\partial \sigma_{r} }}{\partial r} + \frac{{\sigma_{r} - \sigma_{\theta } }}{r} = 0.$$

It is convenient to introduce the following dimensionless quantities

15 $$\rho = \frac{r}{{b_{0} }},\quad a = \frac{{a_{c} }}{{b_{0} }},\quad b = \frac{{b_{c} }}{{b_{0} }},\quad \beta = \frac{{a_{0} }}{{b_{0} }}.$$

## Analytic treatment

By definition, $u_{0} = {{db_{c} } \mathord{\left/ {\vphantom {{db_{c} } {dt}}} \right. \kern-0pt} {dt}}$ and $u = {{dr} \mathord{\left/ {\vphantom {{dr} {dt}}} \right. \kern-0pt} {dt}}$. Therefore, using Eq. (15)

16 $$u = u_{0} \frac{d\rho }{db}.$$

Since $\xi_{r} = {{\partial u} \mathord{\left/ {\vphantom {{\partial u} {\partial r}}} \right. \kern-0pt} {\partial r}}$ and $\xi_{\theta } = {u \mathord{\left/ {\vphantom {u r}} \right. \kern-0pt} r}$, Eq. (8) becomes ${{\partial u} \mathord{\left/ {\vphantom {{\partial u} {\partial r}}} \right. \kern-0pt} {\partial r}} + {u \mathord{\left/ {\vphantom {u r}} \right. \kern-0pt} r} = 0.$ Integrating this equation and using the boundary condition (2) lead to $u = u_{0} b_{c} r^{ - 1}$ or using Eq. (15) to

17 $$\frac{u}{{u_{0} }} = \frac{b}{\rho }.$$

Equations (16) and (17) combine to give ${{d\rho } \mathord{\left/ {\vphantom {{d\rho } {db}}} \right. \kern-0pt} {db}} = b\rho^{ - 1}$. Integrating this equation yields

18 $$\rho^{2} = b^{2} + R^{2} - 1.$$

Here *R* is a Lagrangian coordinate and $\rho = R$ at $b = 1$ (or $b_{c} = b_{0}$). It follows from Eq. (18) that

19 $$\frac{\partial R}{\partial \rho } = \frac{\rho }{R}.$$

Using Eqs. (9), (15), (17) and (18), Eq. (3) can be rewritten in the Lagrangian coordinates as

20 $$\frac{{\partial \varepsilon_{eq} }}{\partial b} = \frac{2}{\sqrt 3 }\frac{b}{{\left( {b^{2} + R^{2} - 1} \right)}}.$$

Integrating this equation and using the initial condition (12) yield

21 $$\varepsilon_{eq} = \frac{1}{\sqrt 3 }\ln \left( {\frac{{b^{2} + R^{2} - 1}}{{R^{2} }}} \right).$$

Equations (5) and (6) combine to give

22 $$\sigma_{\theta } - \sigma_{r} = \frac{{2\sigma_{0} }}{\sqrt 3 }{\Phi} \left( {\varepsilon_{eq} } \right)\Lambda \left( {\alpha_{1} , \ldots ,\alpha_{k} } \right).$$

Using Eqs. (15), (18), (19) and (22), Eq. (14) can be transformed to

23 $$\frac{{\partial \sigma_{r} }}{\partial R} = \frac{{2\sigma_{0} }}{\sqrt 3 }\frac{{{\Phi} \left( {\varepsilon_{eq} } \right)\Lambda \left( {\alpha_{1} , \ldots ,\alpha_{k} } \right)R}}{{\left( {b^{2} + R^{2} - 1} \right)}}.$$

The equivalent strain in this equation should be eliminated by means of Eq. (21). Therefore, the right hand side of Eq. (23) is a function of *b*, *R* and $$\alpha_{p}$$$$(1 \le p \le k).$$ In the Lagrangian coordinates Eq. (10) is

24 $$\frac{{\partial \alpha_{p} }}{\partial b} = \frac{2}{\sqrt 3 }F\left( {\frac{\sigma }{{\sigma_{eq} }},\varepsilon_{eq} ,\alpha_{1} , \ldots ,\alpha_{k} } \right)\frac{b}{{\left( {b^{2} + R^{2} - 1} \right)}},\quad 1 \le p \le k.$$

Here Eqs. (9), (15), (17) and (18) have been used. The first argument of the function *F* is determined from Eqs. (5), (6) and (11) as

25 $$\frac{\sigma }{{\sigma_{eq} }} = \frac{{\sigma_{r} }}{{\sigma_{0} {\Phi} \left( {\varepsilon_{eq} } \right)\Lambda \left( {\alpha_{1} , \ldots ,\alpha_{k} } \right)}} + \frac{1}{\sqrt 3 }.$$

The equivalent strain in Eqs. (24) and (25) should be eliminated by means of Eq. (21). Therefore, the right hand side of Eq. (24) is a function of *b*, *R*, $$\sigma_{r}$$ and $$\alpha_{p}$$$$(1 \le p \le k)$$. Thus Eqs. (23) and (24) constitute the system of equations for $$\sigma_{r}$$ and $$\alpha_{p}$$ in the Lagrangian coordinates. It is evident that the characteristic curves of this system are $$R = {\text{constant}}$$ and $$b = {\text{constant}}$$. The boundary condition (1) becomes

26 $$\sigma_{r} = 0$$

for $R = 1$. Using this condition Eq. (24) can be integrated along the characteristic curve $R = 1$. In particular, substituting Eq. (26) into Eqs. (21) and (25) and, then, the resulting expressions for $\sigma_{r}$ and $\varepsilon_{eq}$ into Eq. (24) yield

27 $$b\frac{{d\alpha_{p} }}{db} = \frac{2}{\sqrt 3 }F\left( {\frac{1}{\sqrt 3 },\;\frac{2}{\sqrt 3 }\ln b,\;\alpha_{1} , \ldots ,\alpha_{k} } \right),\quad 1 \le p \le k$$

The initial conditions to these equations follow from Eq. (13) in the form

28 $$\alpha_{p} = \alpha_{p0} \;\left( {1 \le p \le k} \right)$$

for $b = 1$. It is evident that Eq. (27) can be solved for any given function *F*. Analogously, Eq. (23) can be integrated along the characteristic curve $b = 1$. In particular, using Eqs. (12) and (13)

$$\frac{{d\sigma_{r} }}{dR} = \frac{{2\sigma_{0} }}{\sqrt 3 }\frac{{\Lambda \left( {\alpha_{10} , \ldots ,\alpha_{k0} } \right)}}{R}.$$

Integrating this equation and using the boundary condition (26) yield

29 $$\frac{{\sigma_{r} }}{{\sigma_{0} }} = \frac{2}{\sqrt 3 }\Lambda \left( {\alpha_{10} , \ldots ,\alpha_{k0} } \right)\ln R.$$

The solution of Eqs. (27) and (29) facilitate a numerical treatment of Eqs. (23) and (24).

## Numerical treatment

A finite difference method is adopted to solve Eqs. (23) and (24). *N* + 1 nodes are chosen on the spatial coordinate and *T* + 1 nodes on the time-like coordinate. Therefore, the mesh used is

$$R_{j} = \beta +\Delta R\left( {j - 1} \right),\quad \Delta R = \frac{1 - \beta }{N},\quad 1 \le j \le N + 1,$$

30 $$b_{i} = 1 + \Delta b\left( {i - 1} \right),\quad \Delta b = \frac{{b_{m} - 1}}{T},\quad 1 \le i < T + 1.$$

The value of $\beta$ has been introduced in Eq. (15) and the value of $b_{m}$ should be prescribed.

The calculation starts from the point $j = N$ and $i = 2$. The first approximation to the values of $\sigma_{r}$ and $\alpha_{p}$ at this point is determined as

31 $$\left. {\sigma_{r} } \right|_{N,2}^{pr} = \left. {\sigma_{r} } \right|_{N + 1,2} - \Delta R\left. {\frac{{\partial \sigma_{r} }}{\partial R}} \right|_{N + 1,2} ,\quad \left. {\alpha_{p} } \right|_{N,2}^{pr} = \left. {\alpha_{p} } \right|_{N,1} + \Delta b\left. {\frac{{\partial \alpha_{p} }}{\partial b}} \right|_{N,1} ,\quad 1 \le p \le k,$$

where the derivatives can be found from Eqs. (23) and (24) with no difficulty since the distributions of $\sigma_{r}$ and $\alpha_{p}$ along the lines $j = N + 1$ and $i = 1$ are known from Eqs. (26), (28), (29) and the solution of Eq. (27). Having the values of $\sigma_{r} |_{N,2}^{pr}$ and $\alpha_{p} |_{N,2}^{pr}$ and using Eqs. (23) and (24) the derivatives ${{\partial \sigma_{r} } \mathord{\left/ {\vphantom {{\partial \sigma_{r} } {\partial R}}} \right. \kern-0pt} {\partial R}}$ and ${{\partial \alpha_{p} } \mathord{\left/ {\vphantom {{\partial \alpha_{p} } {\partial b}}} \right. \kern-0pt} {\partial b}}$ can be found at the point $j = N$ and $i = 2$, $\left( {{{\partial \sigma_{r} } \mathord{\left/ {\vphantom {{\partial \sigma_{r} } {\partial R}}} \right. \kern-0pt} {\partial R}}} \right)|_{N,2}$ and $\left( {{{\partial \alpha_{p} } \mathord{\left/ {\vphantom {{\partial \alpha_{p} } {\partial b}}} \right. \kern-0pt} {\partial b}}} \right)|_{N,2}$, $1 \le p \le k$. Then, the derivatives within the intervals $1 - \Delta R \le R \le 1$ and $1 \le b \le 1 + \Delta b$ are approximated by

32 $$\left. {\frac{{\partial \sigma_{r} }}{\partial R}} \right|_{2}^{N + 1,N} = \frac{1}{2}\left( {\left. {\frac{{\partial \sigma_{r} }}{\partial R}} \right|_{N + 1,2} + \left. {\frac{{\partial \sigma_{r} }}{\partial R}} \right|_{N,2} } \right),\quad \left. {\frac{{\partial \alpha_{p} }}{\partial b}} \right|_{1,2}^{N} = \frac{1}{2}\left( {\left. {\frac{{\partial \alpha_{p} }}{\partial b}} \right|_{N,1} + \left. {\frac{{\partial \alpha_{p} }}{\partial b}} \right|_{N,2} } \right),\quad 1 \le p \le k.$$

Replacing the derivatives in Eq. (31) by the values given in Eq. (32) a more accurate approximation of $\sigma_{r}$ and $\alpha_{p}$ at the point $j = N$ and $i = 2$ is determined. It is obvious that this procedure can be extended to $N - 1 \ge j \ge 1.$ As a result, the distribution of $\sigma_{r}$ and $\alpha_{p}$ along the line $i = 2$ is obtained. The line $i = 3$ can be treated in a similar manner since the solution of Eq. (27) is available for any *b*. This procedure can be extended to any number *i*.

## Illustrative example

Assume that $k = 1$, $\alpha_{1} = D$ and $\alpha_{10} = D_{0}$ where *D* is a damage parameter and $D_{0}$ is its initial value. In this case typical functions *F* and ${\Phi}$ are given by Hartley et al. (1997)

$$\begin{aligned} F\left( {\frac{\sigma }{{\sigma_{eq} }},\;\varepsilon_{eq} ,\;D} \right) = q\exp \left( {\frac{3}{2}\frac{\sigma }{{\sigma_{eq} }}} \right)\varepsilon_{eq}^{{{2 \mathord{\left/ {\vphantom {2 M}} \right. \kern-0pt} M}}} , \hfill \\ \hfill \\ \end{aligned}$$

33 $${\Phi} \left( {\varepsilon_{eq} } \right) = 1 + A\varepsilon_{eq}^{{{1 \mathord{\left/ {\vphantom {1 M}} \right. \kern-0pt} M}}} ,$$

where *M* is the Ramberg–Osgood hardening exponent, and $q$ and *A* are material constants. Then, Eq. (27) becomes

$$b\frac{dD}{db} = \frac{2q}{\sqrt 3 }\left( {\frac{2}{\sqrt 3 }} \right)^{{{2 \mathord{\left/ {\vphantom {2 M}} \right. \kern-0pt} M}}} \exp \left( {\frac{\sqrt 3 }{2}} \right)\left( {\ln b} \right)^{{{2 \mathord{\left/ {\vphantom {2 M}} \right. \kern-0pt} M}}} .$$

Integrating this equation and using the initial condition (28) result in

34 $$D = \frac{2qM}{{\sqrt 3 \left( {2 + M} \right)}}\left( {\frac{2}{\sqrt 3 }} \right)^{{{2 \mathord{\left/ {\vphantom {2 M}} \right. \kern-0pt} M}}} \exp \left( {\frac{\sqrt 3 }{2}} \right)\left( {\ln b} \right)^{{{{\left( {2 + M} \right)} \mathord{\left/ {\vphantom {{\left( {2 + M} \right)} M}} \right. \kern-0pt} M}}} + D_{0}$$

at $$R = 1$$.

The values $M = 2.4$, $A = 1.52$ and $q = 1$ were used in all calculations. These values are representative for 2024-T351 aluminum alloy (Hartley et al. 1997). It was initially assumed that $N = 100$ and $T = 200$. The accuracy of calculations was controlled by solving the boundary value problem at $N = 200$ and $T = 400$. The maximum difference was less than 1 %.

**Fig. 1:**
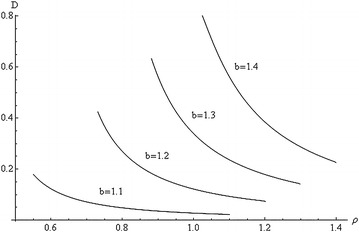
Variation of *D* with *ρ* at $\beta = 0.3$, $q = 1$, and several values of *b*

**Fig. 2:**
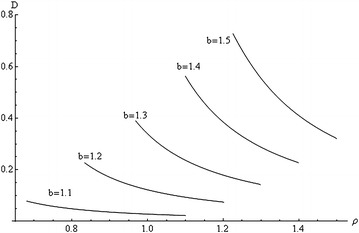
Variation of *D* with *ρ* at $\beta = 0.5$, $q = 1$, and several values of *b*

**Fig. 3:**
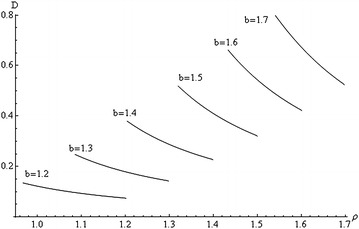
Variation of *D* with *ρ* at $\beta = 0.7$, $q = 1$, and several values of *b*

The fracture criterion is taken as $D = D_{c}$ where $D_{c}$ is constant. The effect of $\beta$ on the evolution of damage at $D_{0} = 0$ and $D_{c} = 0.8$ is illustrated in Figs. 1, 2 and 3. In particular, $\beta = 0.3$ in Fig. 1, $\beta = 0.5$ in Fig. 2 and $\beta = 0.7$ in Fig. 3. In each figure, the distribution of *D* is depicted for several values of *b*. The maximum value of *b* corresponds to the initiation of ductile fracture at the inner radius of the tube.

## Conclusions

The initial/boundary value problem for calculating the evolution of internal variables in an expanding hollow tube at large strains has been reduced to simple equations in characteristic coordinates. An advantage of the approach proposed is that an arbitrary hardening law and an arbitrary set of internal variable evolution equations are included in the formulation. The equilibrium equation has been integrated analytically at $b = 1$ [see Eq. (29)]. The internal variable evolution equations can be integrated analytically or, in the most general case, reduced to numerical integration at $R = 1$ independently of the general solution of the initial/boundary value problem [see Eq. (27)]. These solutions have been used in the numerical scheme proposed. They can also be used in conjunction with any other numerical scheme to increase the accuracy of calculation and to reveal possible errors in numerical solutions.

The illustrative example deals with the evolution of damage. It is seen from Figs. 1, 2 and 3 that the initiation of ductile fracture occurs at the inner radius of the tube in all the cases considered. It is worth noting here that the fracture initiation in an expanding thick ring may occur at mid-annulus (Tomkins and Atkins 1981). It is evident that the damaged model adopted in the present paper is not appropriate in such cases. However, the general solution found and the general numerical scheme developed are independent of the specific form of the functions ${\Phi}$ and *F*. The present general solution can be used to test various function involved in Eqs. (5) and (10) to find functions ${\Phi}$ and *F* that are in agreement with the experimental result presented by Tomkins and Atkins (1981).

The accuracy of the numerical solution is rather high. Therefore, the final result is useful for verifying more general numerical codes, which can be used to solve arbitrary initial/boundary value problems.

## References

[CR1] Alexandrov S, Jeng Y-R (2011). Damage evolution in an expanding/contracting hollow sphere at large strains. Contin Mech Thermodyn.

[CR2] Besson J (2010). Continuum models of ductile fracture: a review. Int J Damage Mech.

[CR3] Bland DR (1956). Elastoplastic thick-walled tubes of work-hardening material subject to internal and external pressures and to temperature gradients. J Mech Phys Solids.

[CR4] Chandrakanth S, Pandey PC (1993). A new ductile damage evolution model. Int J Fract.

[CR5] Chen PCT (1986). The Bauschinger and hardening effect on residual stresses in an autofrettaged thick-walled cylinder. J Press Vessel Technol.

[CR6] Cheong WC, Kam HK, Wang CC, Lim YP (2014). Rigid-plastic finite element simulation of cold forging and sheet metal forming by Eulerian meshing method. Adv Mater Res.

[CR7] Eom J, Chung W, Joun M (2014). Comparison of rigid-plastic and elastoplastic finite element predictions of a tensile test of cylindrical specimens. Key Eng Mater.

[CR8] Farrahi GH, Voyiadjis GZ, Hoseini SH, Hosseinian E (2013). Residual stress analysis of the autofrettaged thick-walled tube using nonlinear kinematic hardening. J Press Vessel Technol.

[CR9] Fatehi P, Nejad MZ (2014). Effects of material gradients on onset of yield in FGM rotating thick cylindrical shells. Int J Appl Mech.

[CR10] Ganapathysubramanian S, Zabaras N (2004). Deformation process design for control of microstructure in the presence of dynamic recrystallization and grain growth mechanisms. Int J Solids Struct.

[CR11] Guo YM, Kamitani S (2010). A rigid-plastic hybrid PCM/FEM for metal forming problems. WSEAS Trans Appl Theor Mech.

[CR12] Hartley P, Hall FR, Chiou JM, Pillinger I, Predeleanu M, Gilormini P (1997). Elastic-plastic finite-element modeling of metal forming with damage evolution. Advanced methods in materials processing defects.

[CR13] He YB, Pan QL, Chen Q, Zhang ZY, Liu XY, Li WB (2012). Modeling of strain hardening and dynamic recrystallization of ZK60 magnesium alloy during hot deformation. Trans Nonferrous Metals Soc China.

[CR14] Helsing J, Jonsson A (2002). On the accuracy of benchmark tables and graphical results in the applied mechanics literature. J Appl Mech.

[CR15] Hill R, Lee EH, Tupper SJ (1947). The theory of combined plastic and elastic deformation with particular reference to a thick tube under internal pressure. Proc R Soc A Math Phys Eng Sci.

[CR16] Hore S, Das SK, Banerjee S, Mukherjee S (2015). Computational modelling of static recrystallization and two dimensional microstructure evolution during hot strip rolling of advanced high strength steel. J Manuf Process.

[CR17] Lazzarin P, Livieri P (1997). Different solutions for stress and strain fields in autofrettaged thick-walled cylinders. Int J Press Vessels Pip.

[CR18] Lemaitre J (1985). A continuous damage mechanics model for ductile fracture. J Eng Mater Technol.

[CR19] Leu SY (2007). Analytical and numerical investigation of strain-hardening viscoplastic thick-walled cylinders under internal pressure by using sequential limit analysis. Comput Methods Appl Mech Eng.

[CR20] Leu SY (2009). Static and kinematic limit analysis of orthotropic strain-hardening pressure vessels involving large deformation. Int J Mech Sci.

[CR21] Leu SY, Li RS (2012). Exact solutions of sequential limit analysis of pressurized cylinders with combined hardening based on a generalized Holder inequality: formulation and validation. Int J Mech Sci.

[CR22] Lin J, Dean TA (2005). Modelling of microstructure evolution in hot forming using unified constitutive equations. J Mater Process Technol.

[CR23] Livieri P, Lazzarin P (2002). Autofrettaged cylindrical vessels and Bauschinger effect: an analytical frame for evaluating residual stress distributions. J Press Vessel Technol.

[CR24] Loghman A, Wahab MA (1994). Loading and unloading of thick-walled cylindrical pressure vessels of strain-hardening material. J Press Vessel Technol.

[CR25] Megahed MM, Abbas AT (1991). Influence of reverse yielding on residual stresses induced by autofrettage. Int J Mech Sci.

[CR26] Rees DWA (1987). A theory of autofrettage with applications to creep and fatigue. Int J Press Vessels Pip.

[CR27] Rees DWA (1990). Autofrettage theory and fatigue life of open-ended cylinders. J Strain Anal Eng Des.

[CR28] Roberts SM, Hall FR, Bael AV, Hartley P, Pillinger I, Sturgess CEN, Houtte PV, Aernoudt E (1992). Benchmark tests for 3-D, elasto-plastic, finite-element codes for the modeling of metal forming processes. J Mater Process Technol.

[CR29] Stacey A, Webster GA (1988). Determination of residual stress distributions in autofrettaged tubing. Int J Press Vessels Pip.

[CR30] Tomkins B, Atkins AG (1981). Crack initiation in expanded fully plastic thick-walled rings and rotating discs. Int J Mech Sci.

[CR31] Wineman A, Min JH (1996). The pressurized cylinder problem for nonlinear viscoelastic materials with a strain clock. Math Mech Solids.

